# Supplementary material for: Regioselective One-Pot Synthesis, Biological Activity and Molecular Docking Studies of Novel Conjugates N-(p-Aryltriazolyl)-1,5-benzodiazepin-2-ones as Potent Antibacterial and Antifungal Agents
Source: Molecules. 2022 Jun 22;27(13):4015. doi: 10.3390/molecules27134015 (PMC9268147; doi:10.3390/molecules27134015)
Supplement: Supplementary file 1 [file molecules-27-04015-s001.zip › molecules-1755058-supplementary.pdf]

Regioselective one-pot synthesis, biological activity and molecular docking studies of novel conjugates *N*-(p-aryltriazolyl)-1,5-benzodiazepin-2-ones as potent antibacterial and antifungal agents

Asma NSIRA[1], Hasan MTIRAOUI[1], Sami CHNITI[1], Hanan Al-GHULIKAH[2,*], Rafik GHARBI[3] and Moncef MSADDEK[1]

[1] Laboratory of Heterocyclic Chemistry Natural Products and Reactivity/CHPNR, Department of Chemistry, Faculty of Science of Monastir, University of Monastir, 5000 Monastir, Tunisia

[2] Department of Chemistry, College of Sciences, Princess Nourah bint Abdulrahman University, P.O. Box 84428, Riyadh 11671, Saudi Arabia

[3] Laboratory of applied chemistry and environment, Department of Chemistry, Faculty of Science of Monastir, University of Monastir, 5000 Monastir, Tunisia

* Correspondence: haalghulikah@pnu.edu.sa; Tel.: +966-11823-6011

Table of contents:

I.The detailed description of NMR 4b……………………………………………………………S$_2$-S$_5$
II. The detailed description of NMR 6b……………………………………………………………S$_6$-S$_{15}$
II.Spectra of HRMS of 4b and 4g……………………………………………………………S$_{16}$-S$_{17}$
II.2 Copies of ^{1}H and ^{13}C NMR ……………………………………………………………S$_{18}$-S$_{29}$

- Thus, the formation of the triazole unit in **4b** chosen as an example was confirmed by the disappearance in the ^{1}H NMR spectra of a triplet (t, H-3'', 2.35 ppm) corresponding to the acetylenic proton in front of the appearance of the characteristic singlet (s, H-5'', 8,05 ppm) which is directly attributed to the proton of the triazole ring.

Moreover the deshielding alkynes functions characteristic signals at 4.30 ppm (d, 2H, CH2-1', *J* = 15.6 Hz), to the signals corresponding to the methylene alpha to the triazole ring at 4.96 ppm (d, 1H, H-1a '', *J* = 14.7 Hz) and 5.27 ppm (d, 1H, H-1b'', *J* = 15 Hz), as can be seen when compared with their displacements in the starting molecules. Note also the presence of a singlet at 3.89 ppm directly attributed to the methoxy group and signals in the aromatic region at (δH 6,98-8,16 ppm, 12H) corresponding to the aromatic protons of both diazepine and the phenyl ring of the azide used.

Additional supports for our assignments arose from the HMBC spectrum which revealed significant long range correlation between the triazolic proton (s, H-5'', 8.01 ppm) and both quaternary carbons C-4'' and C-1''' at 144.6 ppm and 154.2 ppm respectively. Also, the proton H-3''' (7,62 ppm) correlates with carbons OCH_3, C-2''' et C-1''' appearing at 55,6 ppm, 122,0 ppm and 154,2 ppm respectively. Thus came supporting the obtention of the proposed molecular structures.

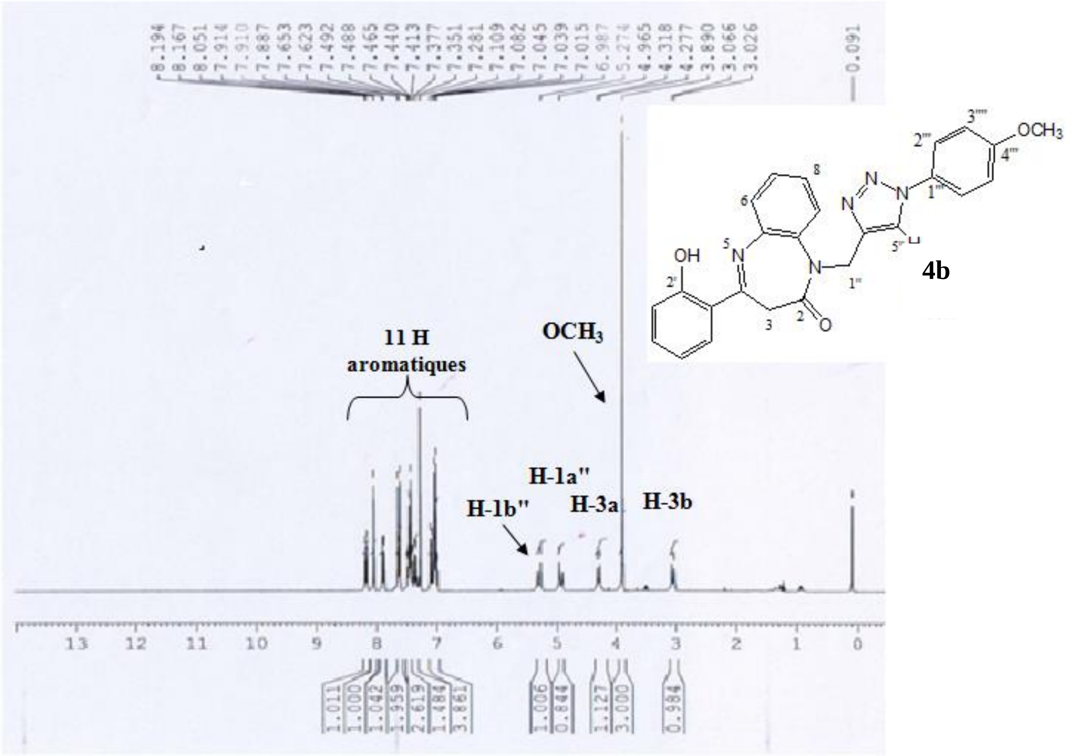

Figure S1 . NMR spectra ^{1}H (300 MHz, $CDCl_3$) of compound **4b**

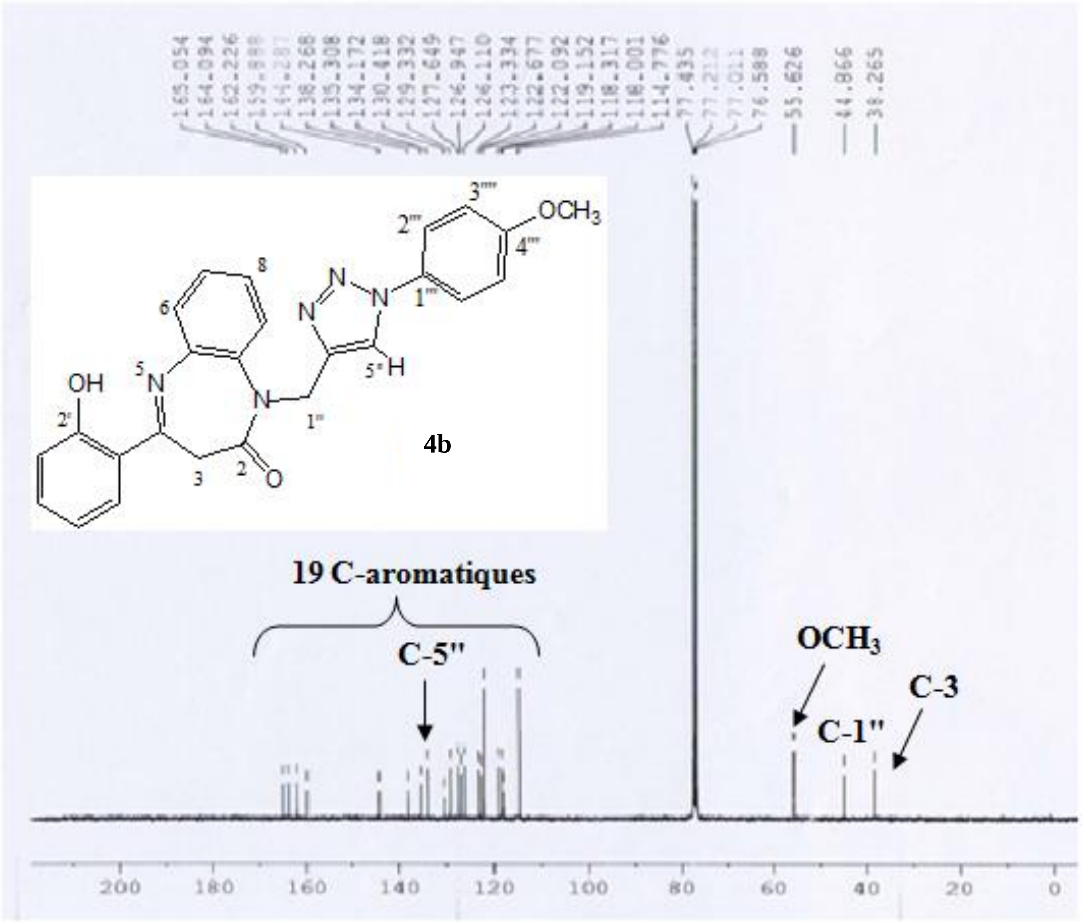

Figure S2 . NMR spectra ^{13}C (75,47 MHz, $CDCl_3$) of compound **4b**

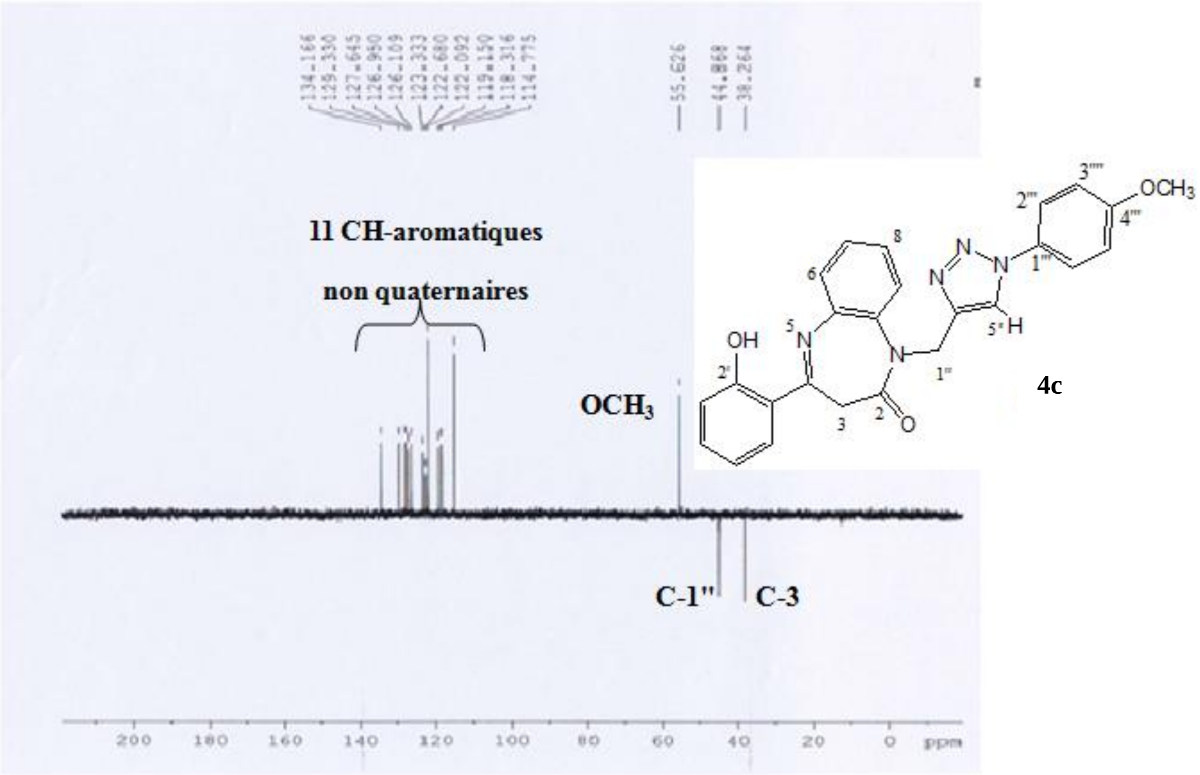

Figure S3 . DEPT 135 of compound **4b**

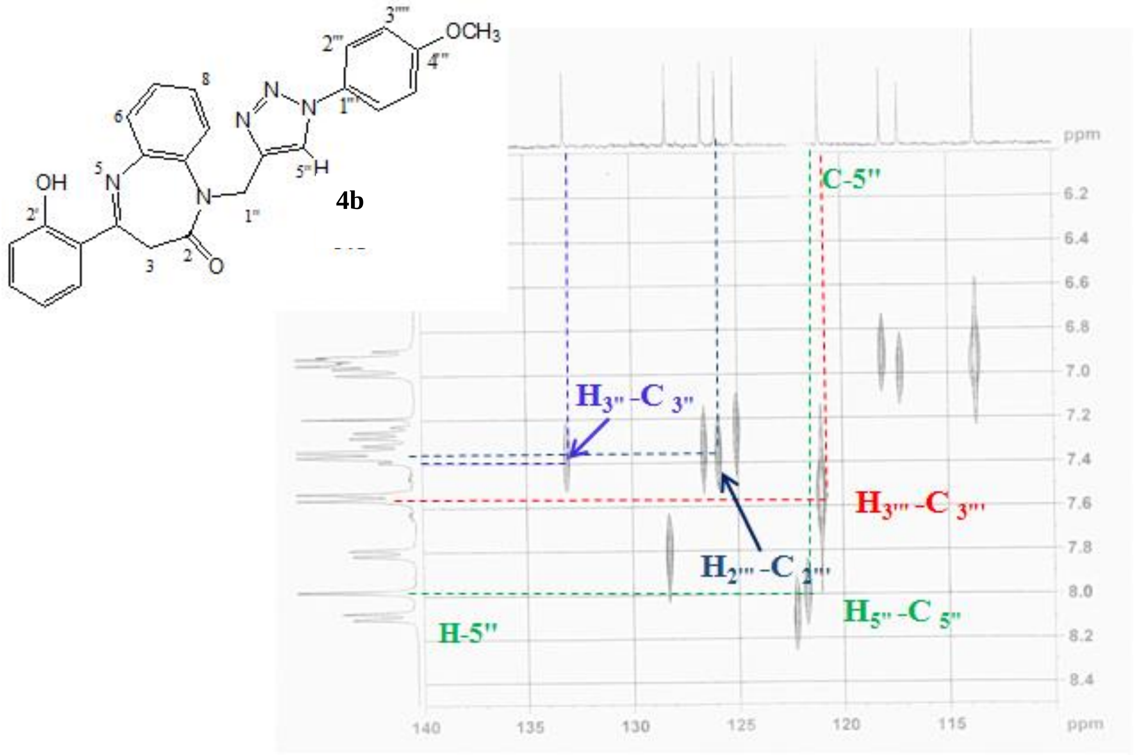

Figure S4 . CHcorr spectra of compound **4b**

- The ^{1}H NMR spectrum of the *N*-galactosyl-triazoles **6b** showed chemical shifts of H-1''' (anomeric proton) at 5.40 ppm (doublet, *J*= 4.8 Hz) downfield compared to the precursor azide at 5.56 ppm.
  Except these characteristic signals taken from the ^{1}H NMR spectrum of compound **6b**, ^{13}C NMR (75.4 MHz in $CDCl_3$) was used and more particularly the DEPT 135 experiment. In addition to the observed peaks for the starting compound, four peaks appeared between 22.6 and 25.9 ppm corresponding to the methyl of the galactopyranosyl part. Moreover, the peaks at δ= 66.8(C-2'''),67.1(C-3'''), 70.2(C-4'''), 70.7(C-5'''), 96.2(C-1'''), 109.9(C-isop), 109.9(C-isop) ppm are assigned to the introduced galactosyl moiety. The peak at 50.56 ppm is attributed to the C-6 '''carbon. A signal at 126.9 ppm is related to carbon C-5 "of the triazole ring.

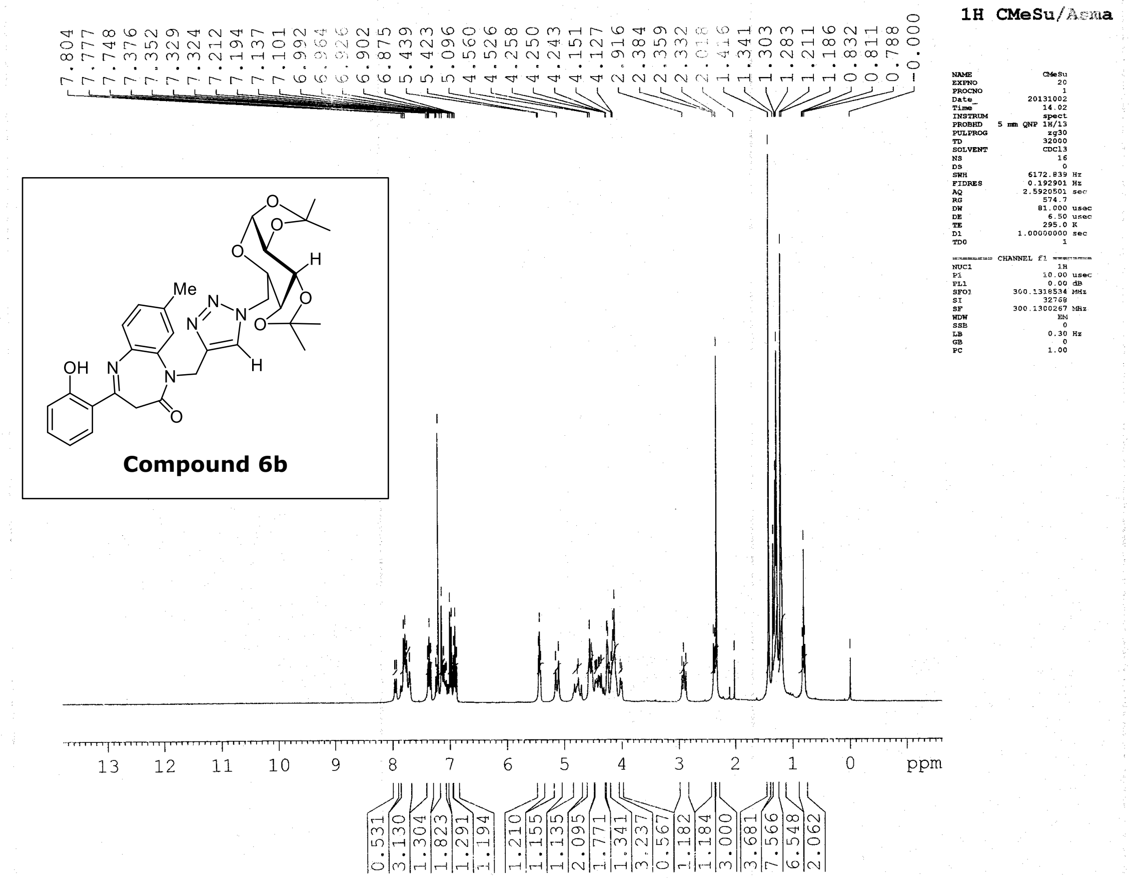

Figure S5. NMR spectra ^{1}H (300 MHz, $CDCl_3$) of compound **6b**

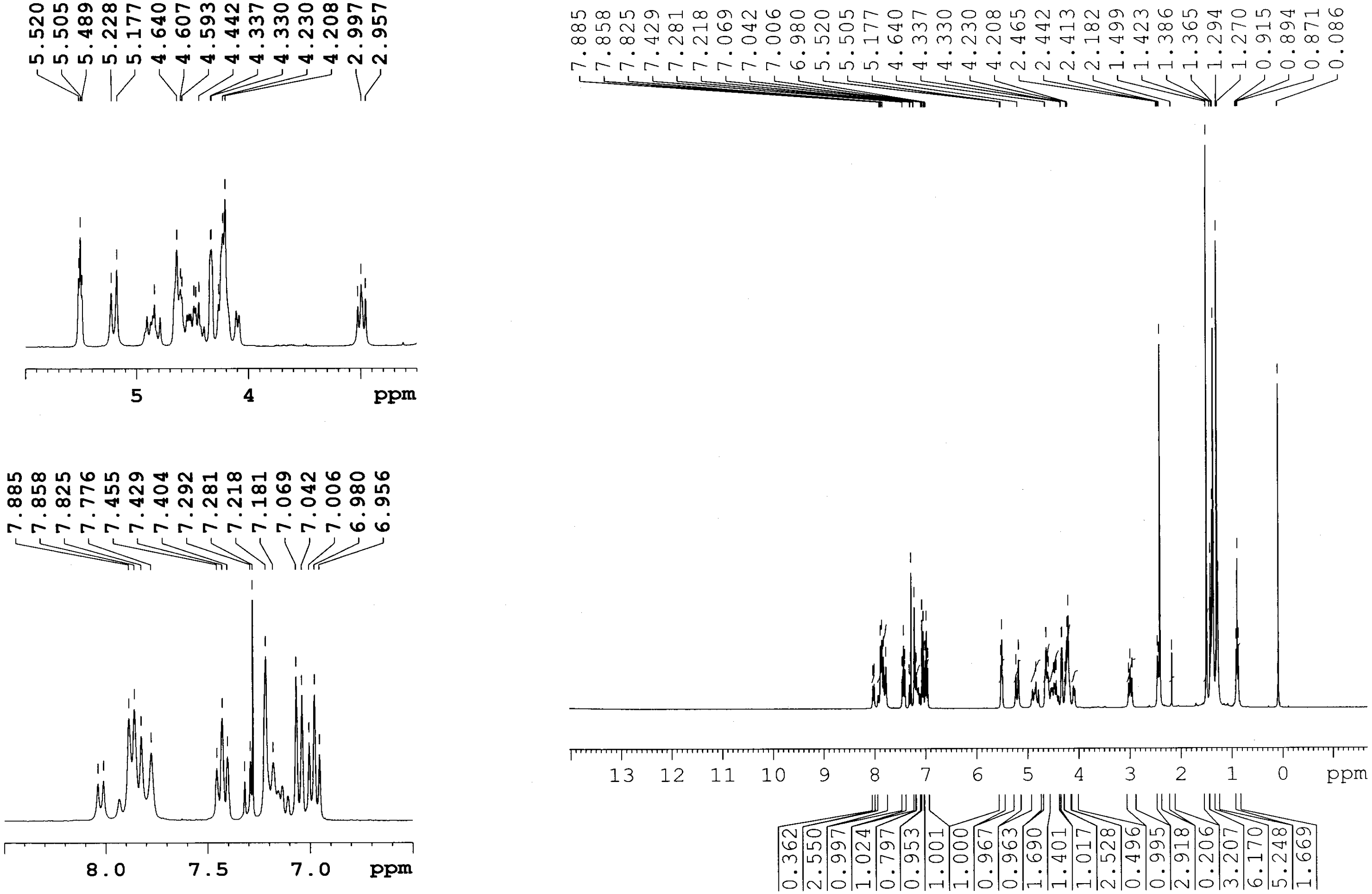

Figure S6. NMR spectra ^{1}H (300 MHz, $CDCl_3$) of compound **6b**

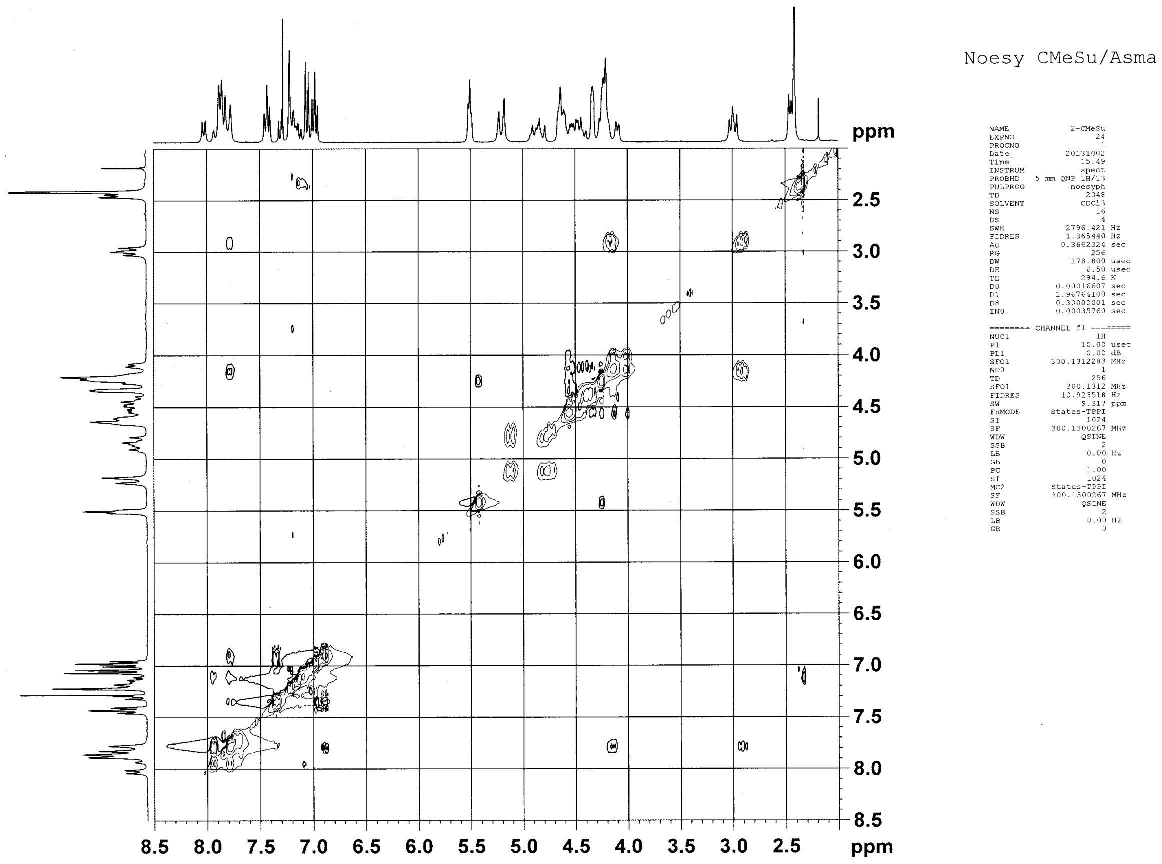

Figure S7. NOESY Spectra of compound **6b**

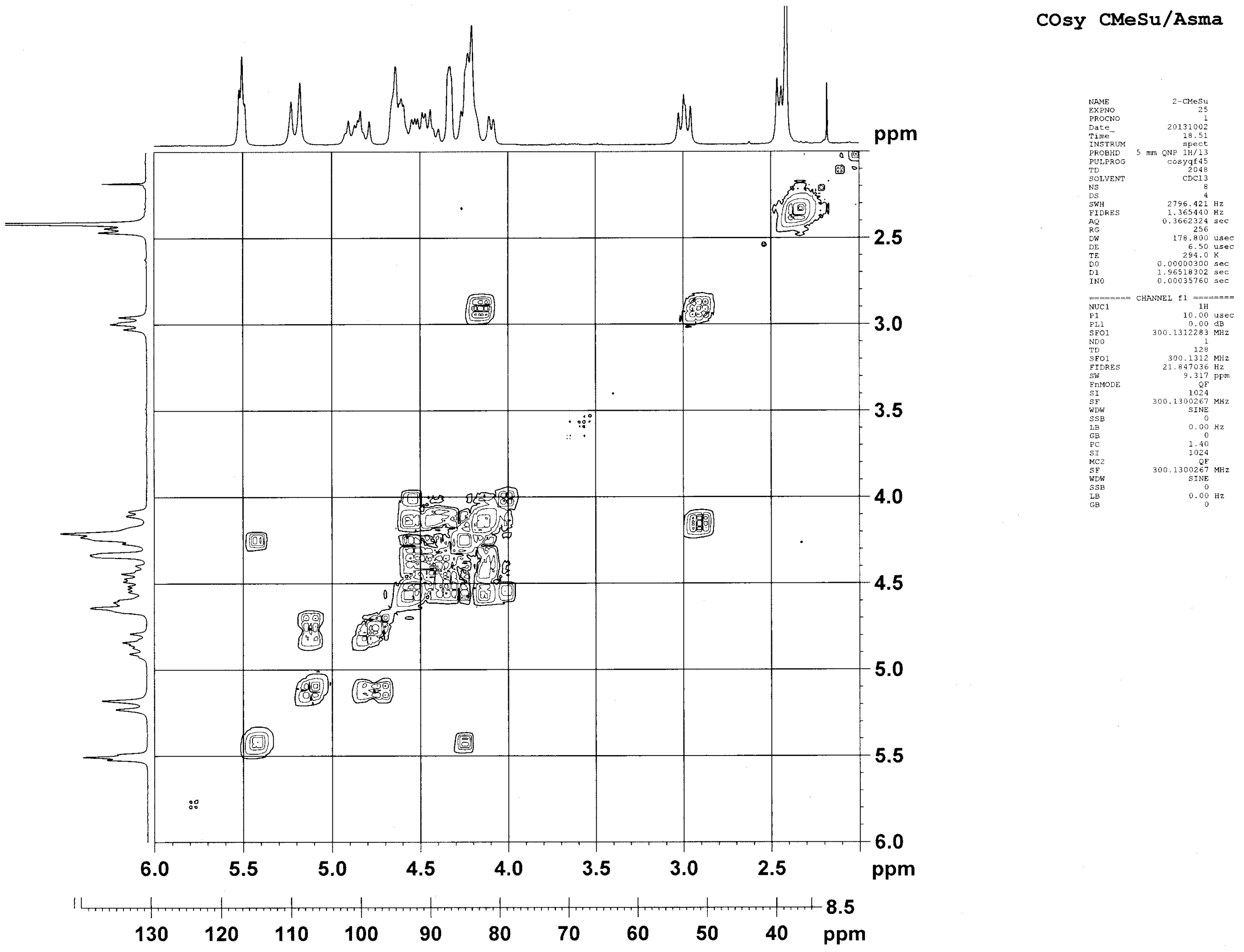

Figure S8 . COSY ^{1}H-^{1}H spectra of compound **6b**

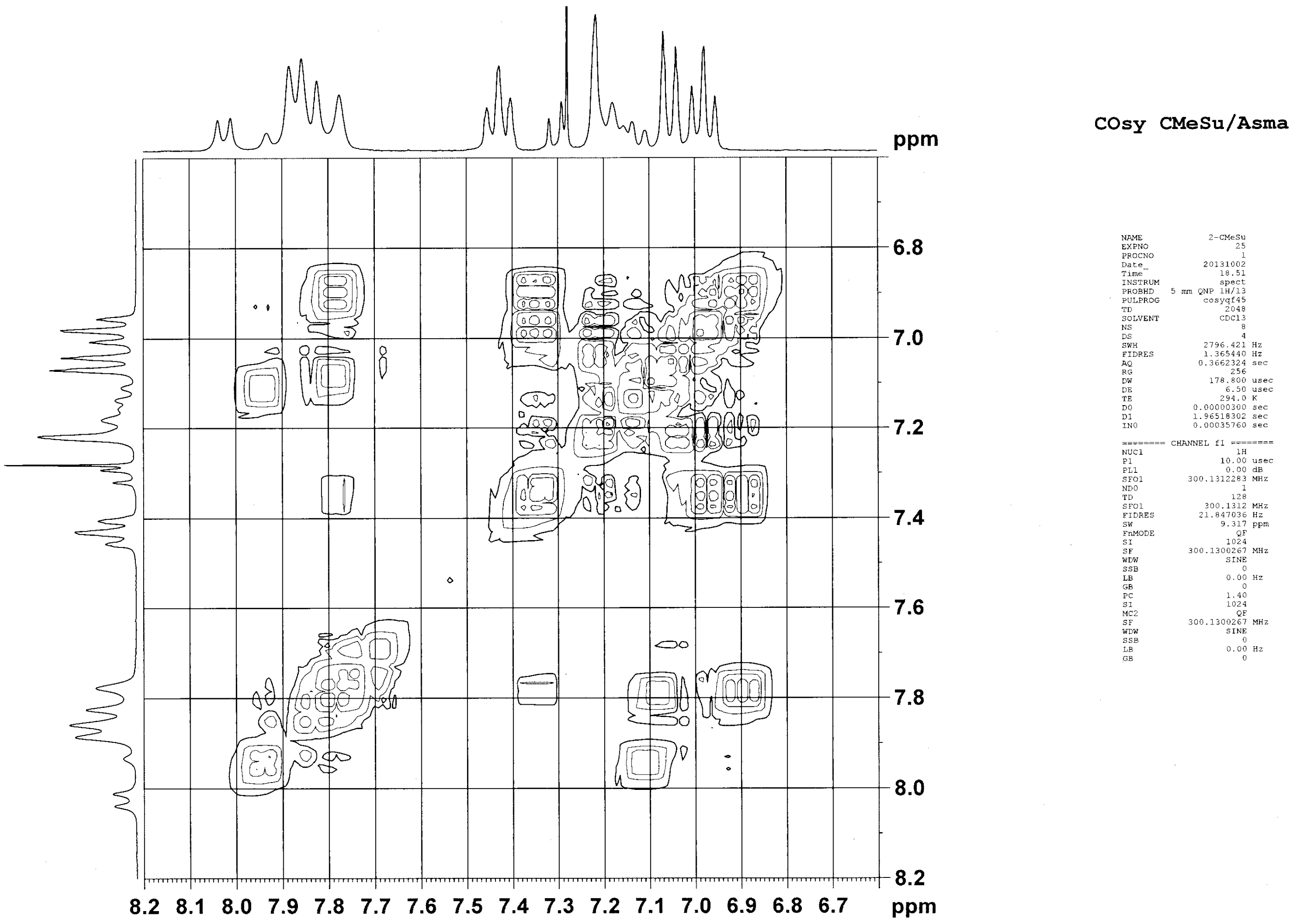

Figure S9 . COSY ^{1}H-^{1}H spectra of compound **6b**

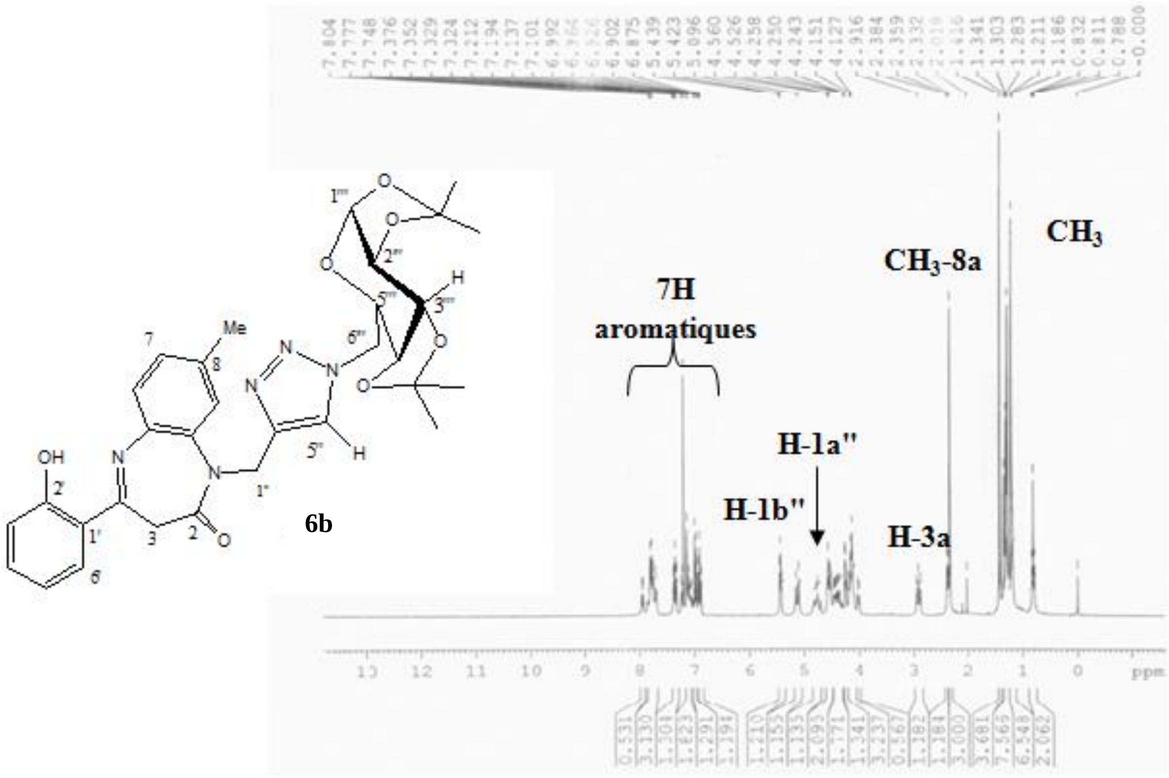

Figure S10. NMR spectra ^{1}H (300 MHz, $CDCl_3$) of compound **6b**

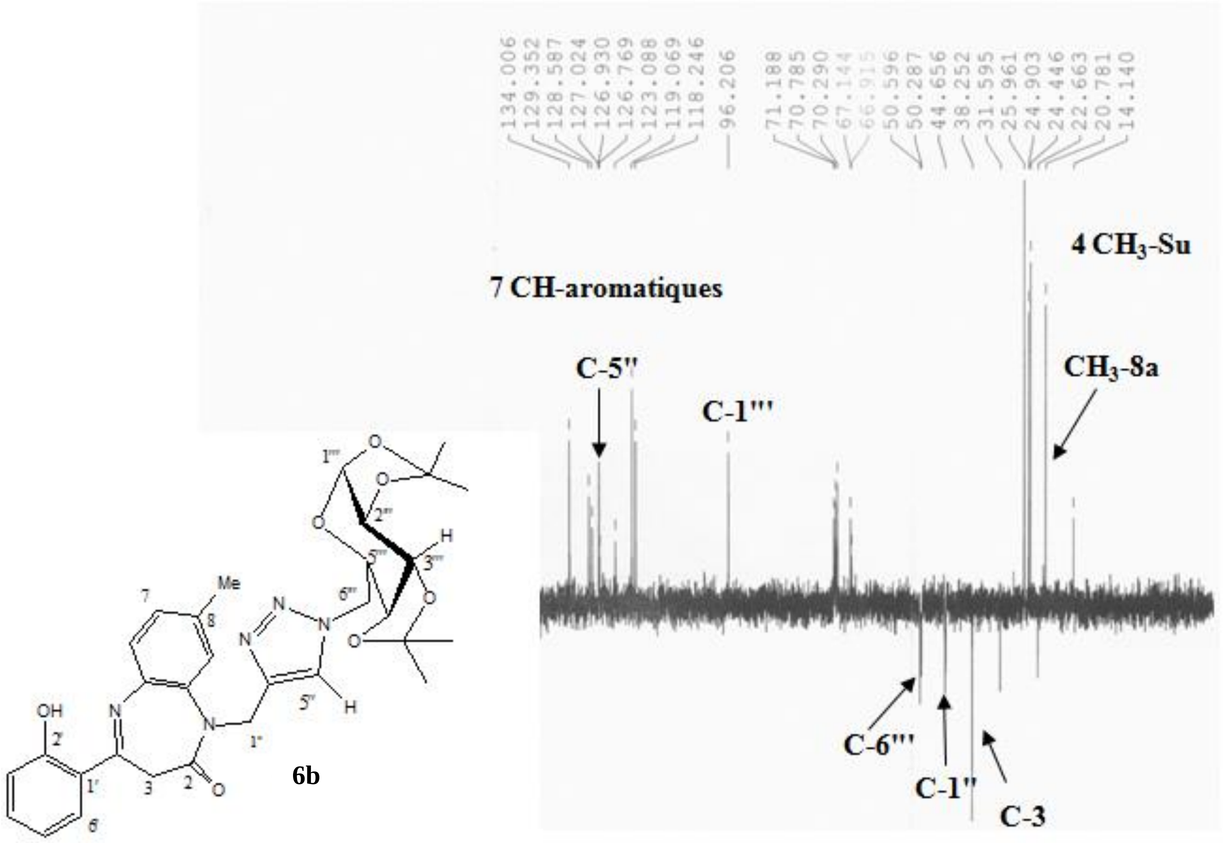

Figure S11. DEPT 135 (75,47 MHz, $CDCl_3$) of compound **6b**

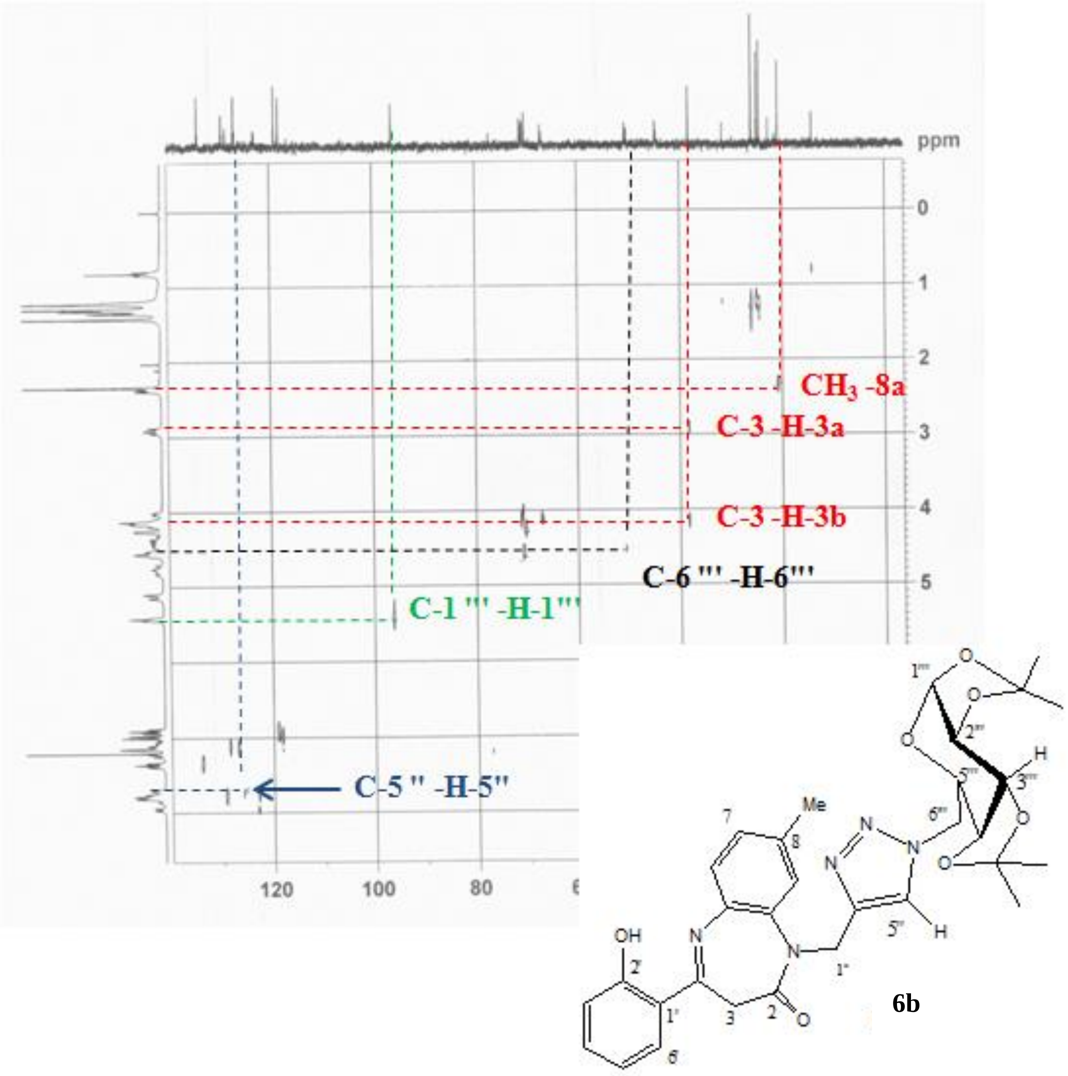

Figure S12 . CHcorr Spectra of compound **6b**

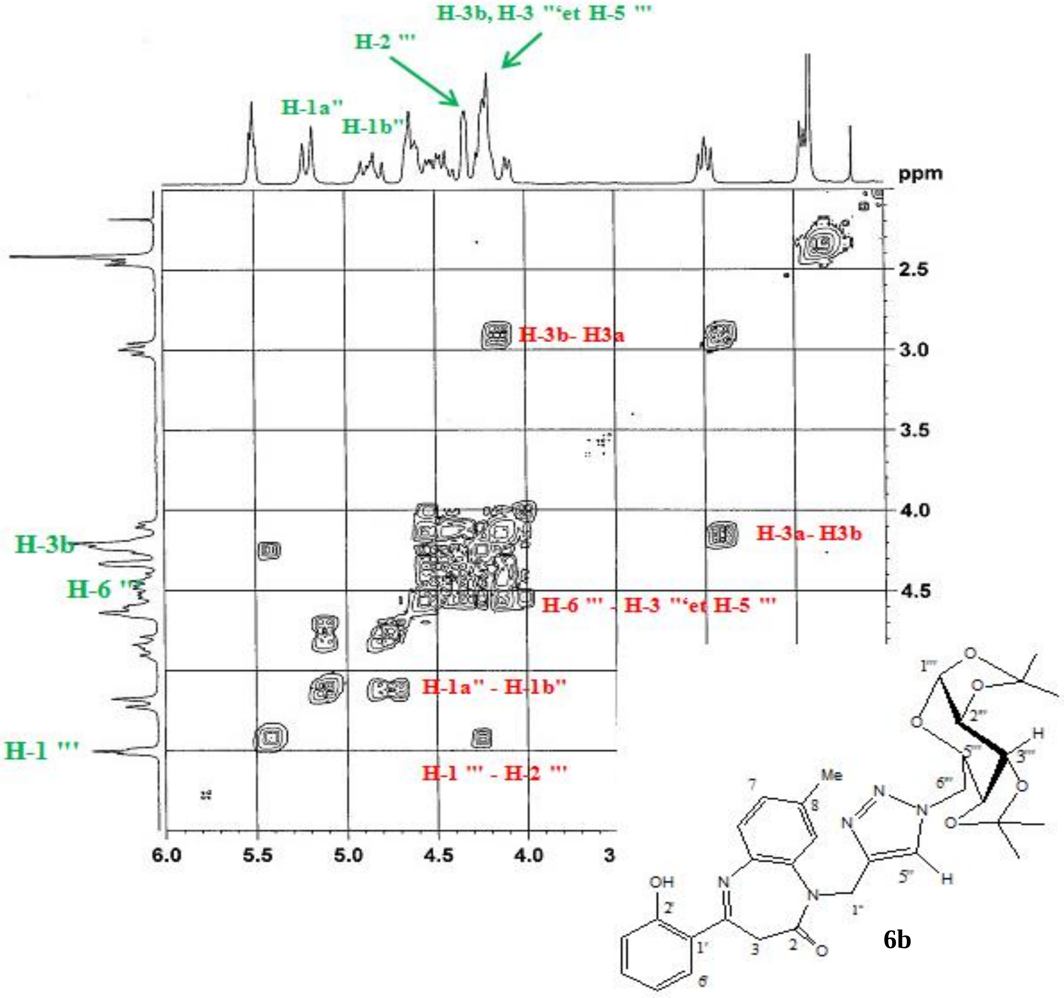

Figure S13. COSY Spectra of compound **6b**

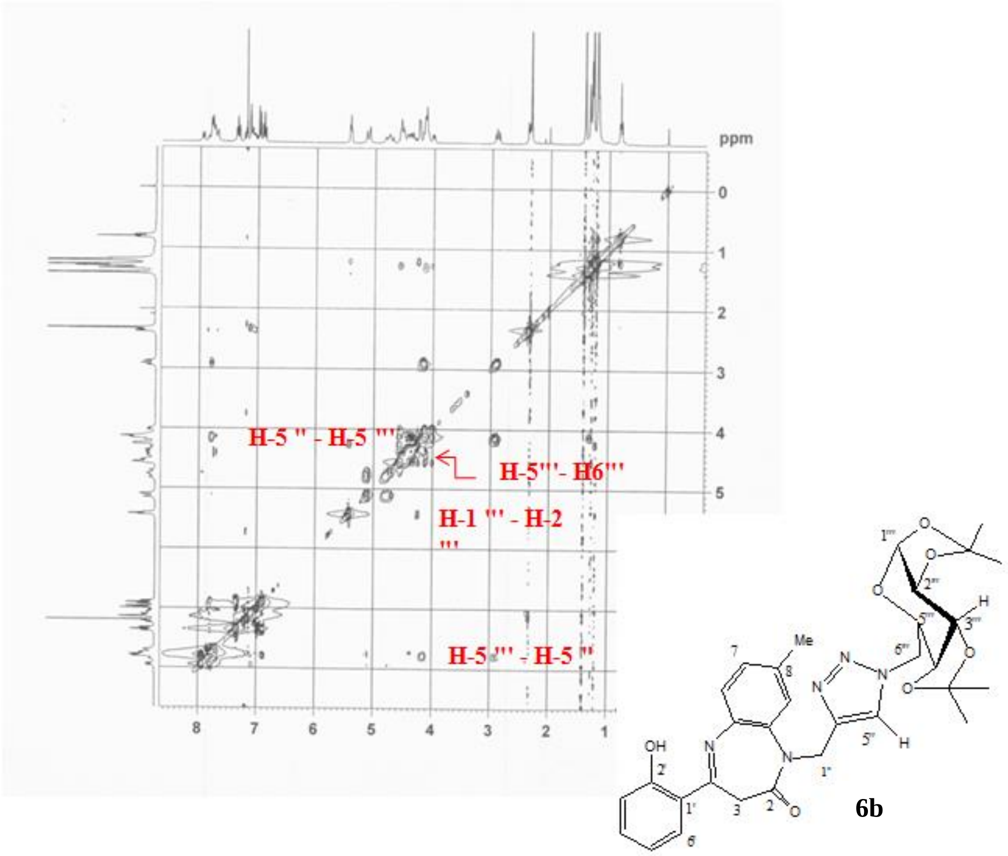

6b

Figure S14. NOESY Spectra of compound **6b**

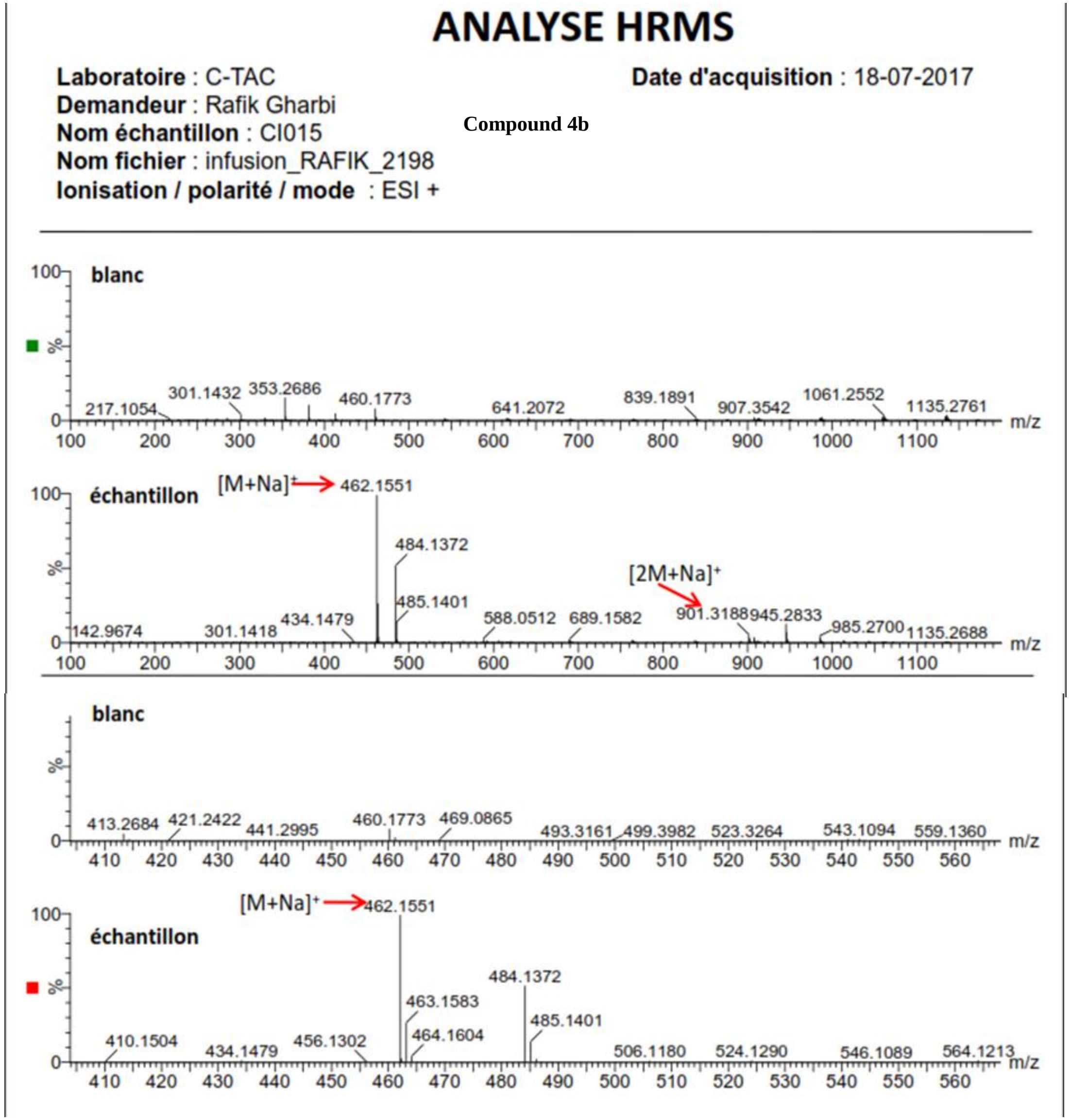

Figure S15. HRMS Spectra of compound **4b**

ANALYSE HRMS

Laboratoire : C-TAC
Demandeur : Rafik Gharbi
Nom échantillon : CI021
Nom fichier : infusion_RAFIK_2202
Ionisation / polarité / mode : ESI +

Date d'acquisition : 18-07-2017

Compound 4g

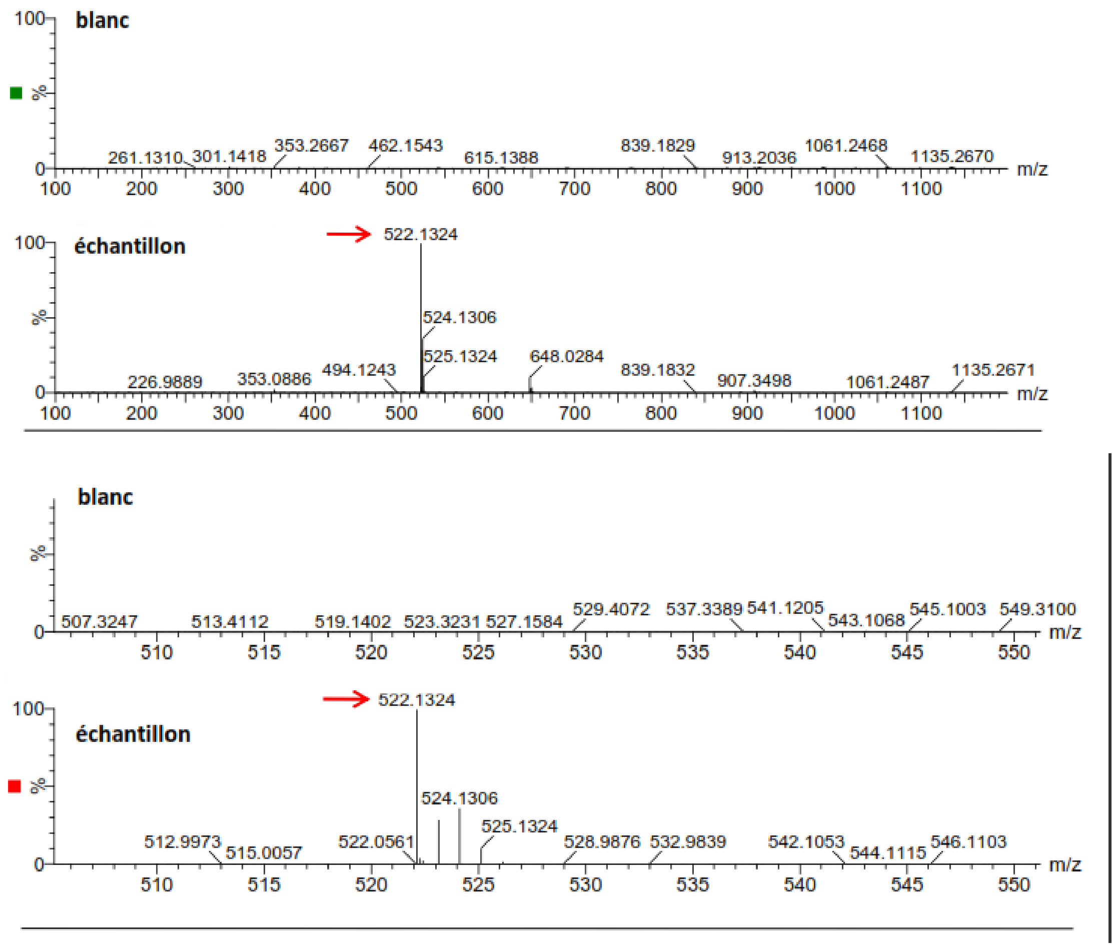

Figure S16. HRMS Spectra of compound **4g**

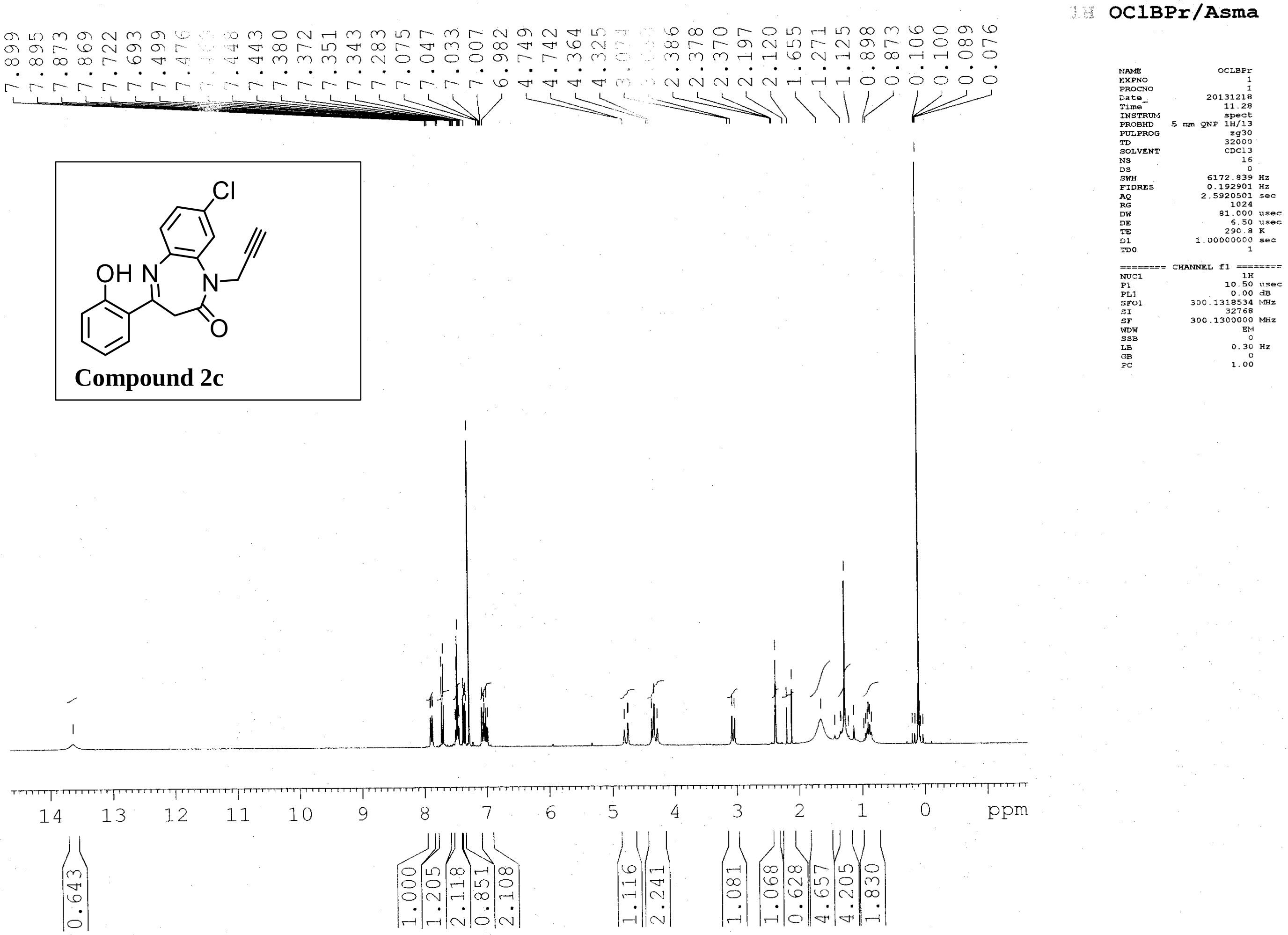

Figure S17. NMR spectra ^{1}H (300 MHz, $CDCl_3$) of compound **2c**

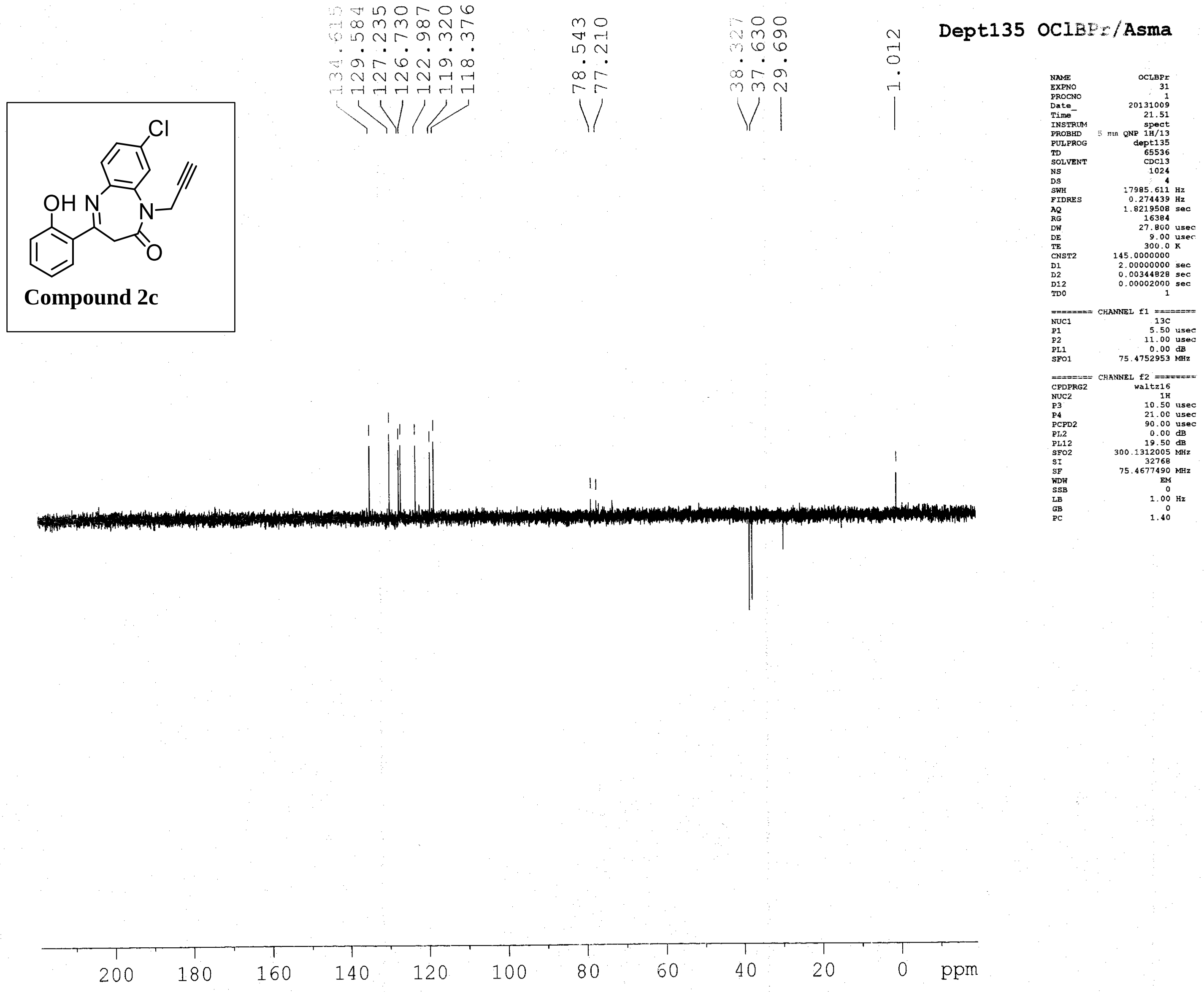

Figure S18 . DEPT 135 of compound **2c**

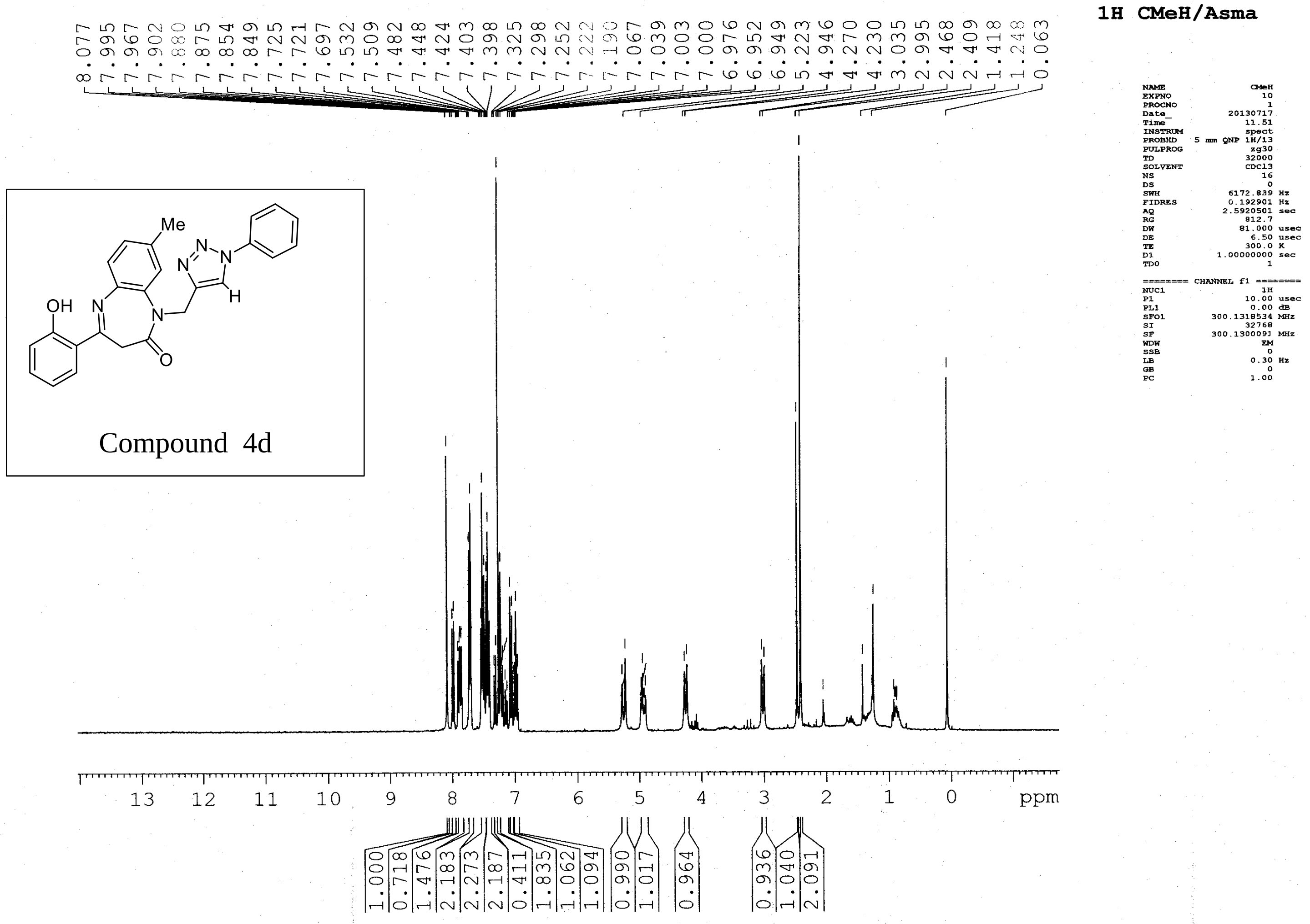

Figure S19. NMR spectra ^{1}H (300 MHz, $CDCl_3$) of compound **4d**

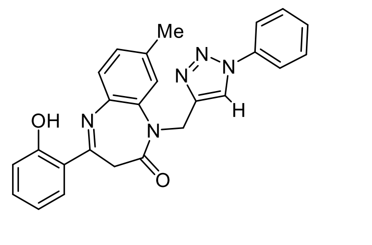
Compound 4d

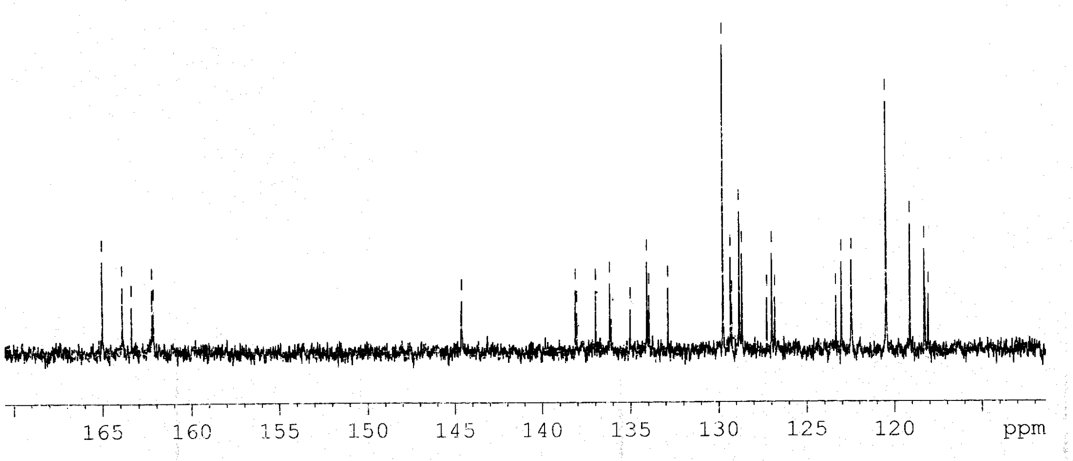

Figure S20. NMR spectra ^{13}C (75,47 MHz, $CDCl_3$) of compound **4d**

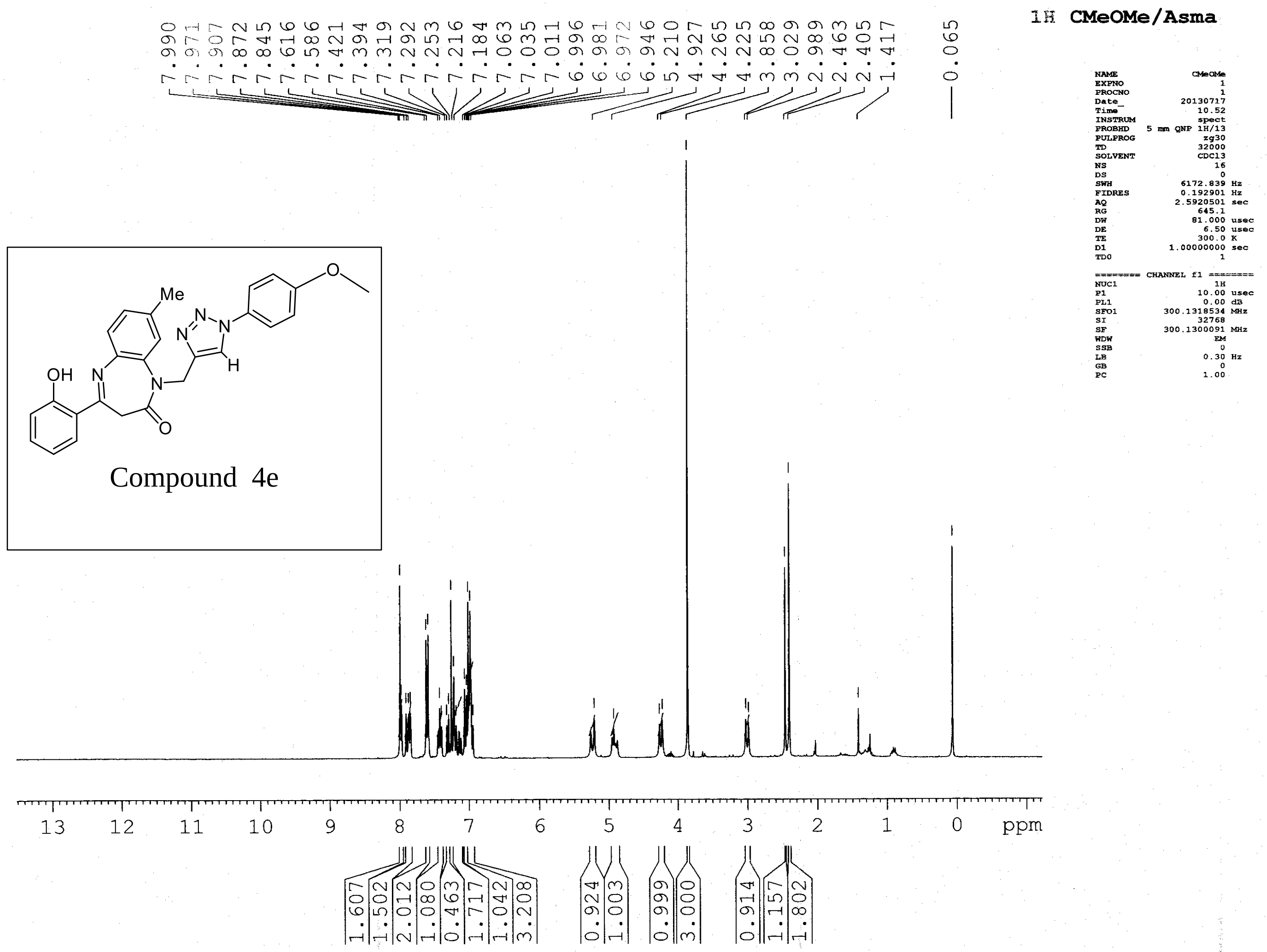

Figure S21. NMR spectra ^{1}H (300 MHz, $CDCl_3$) of compound **4e**

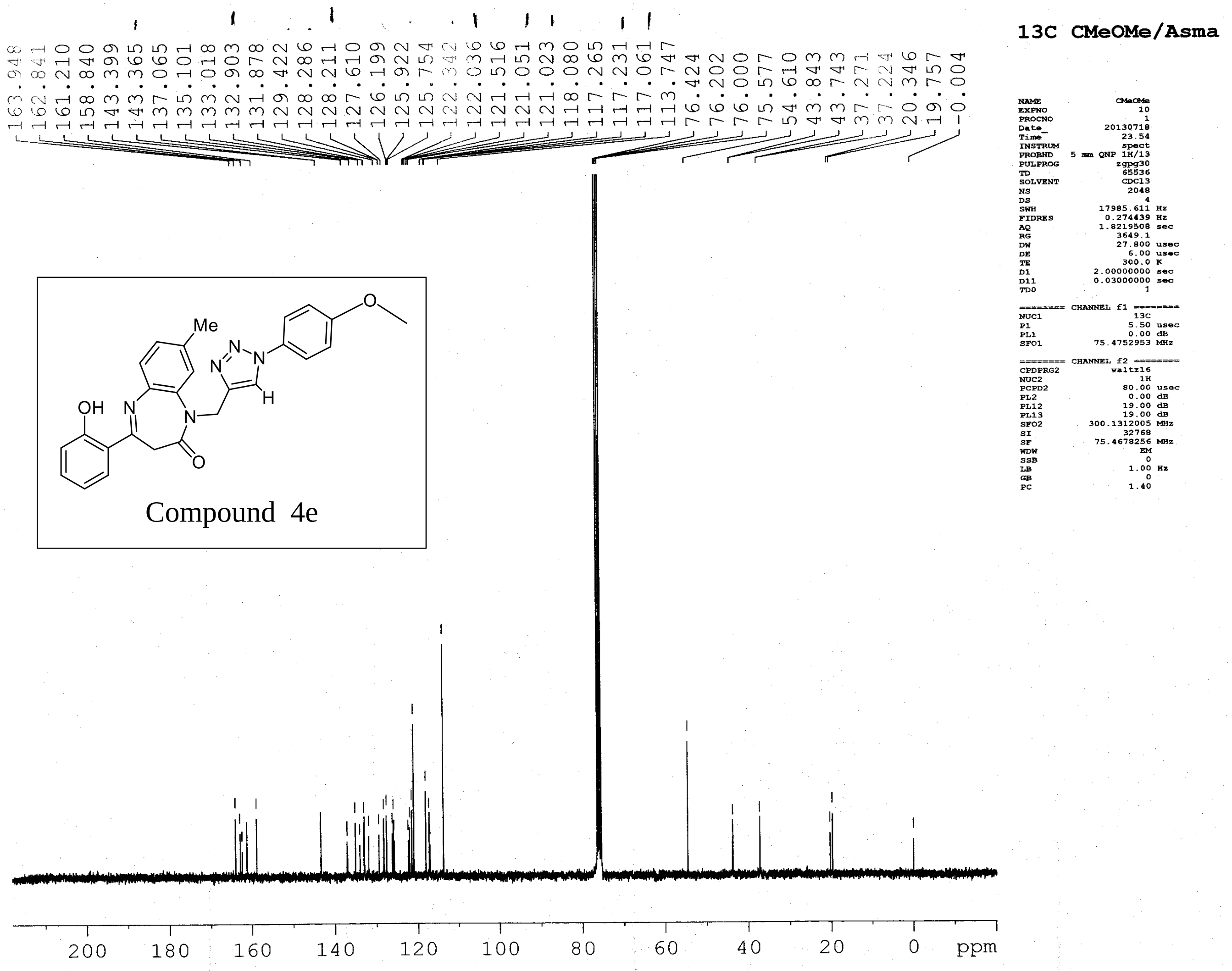

Figure S22. NMR spectra ^{13}C (75,47 MHz, $CDCl_3$) of compound **4e**

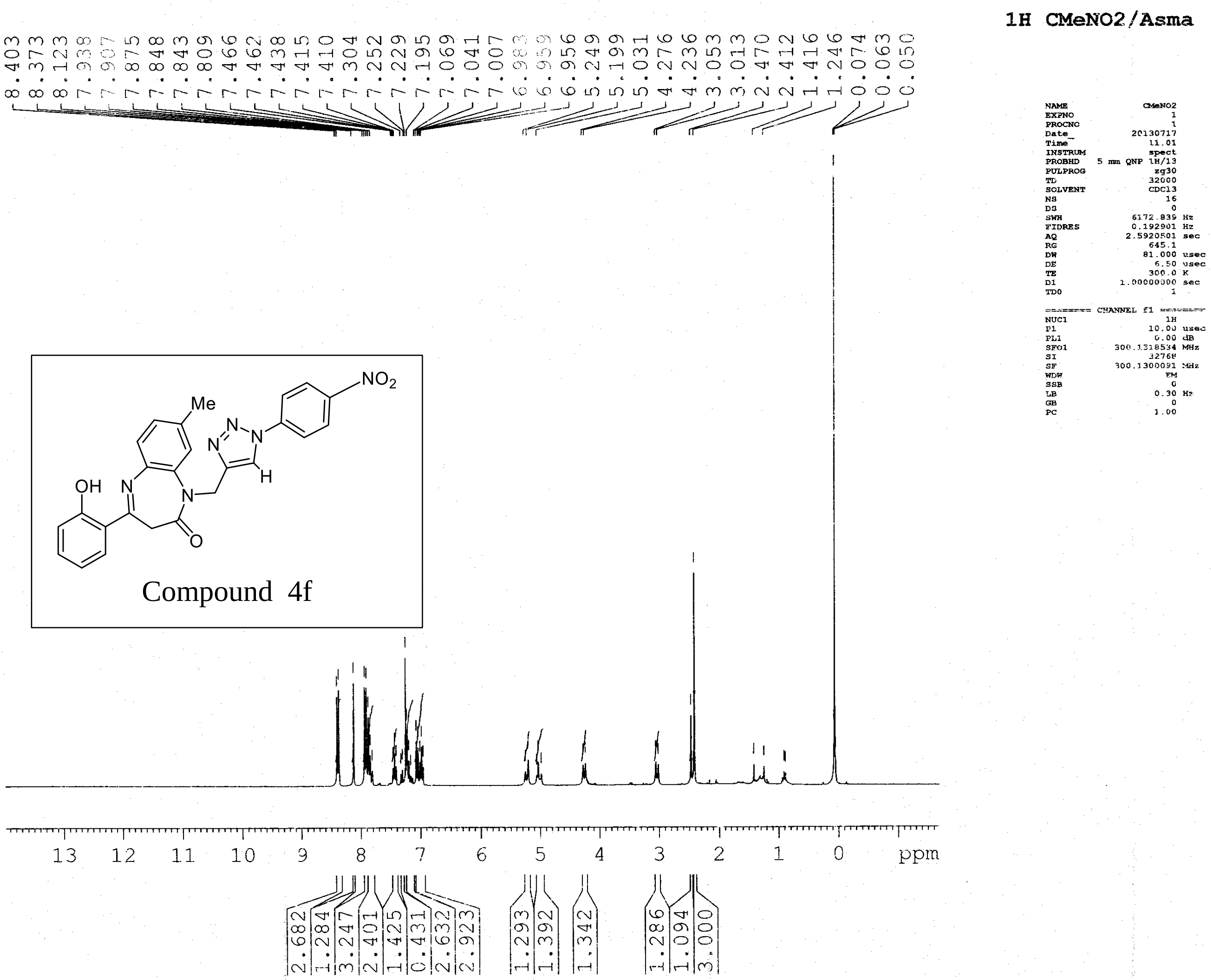

Figure S23. NMR spectra ^{1}H (300 MHz, $CDCl_3$) of compound **4f**

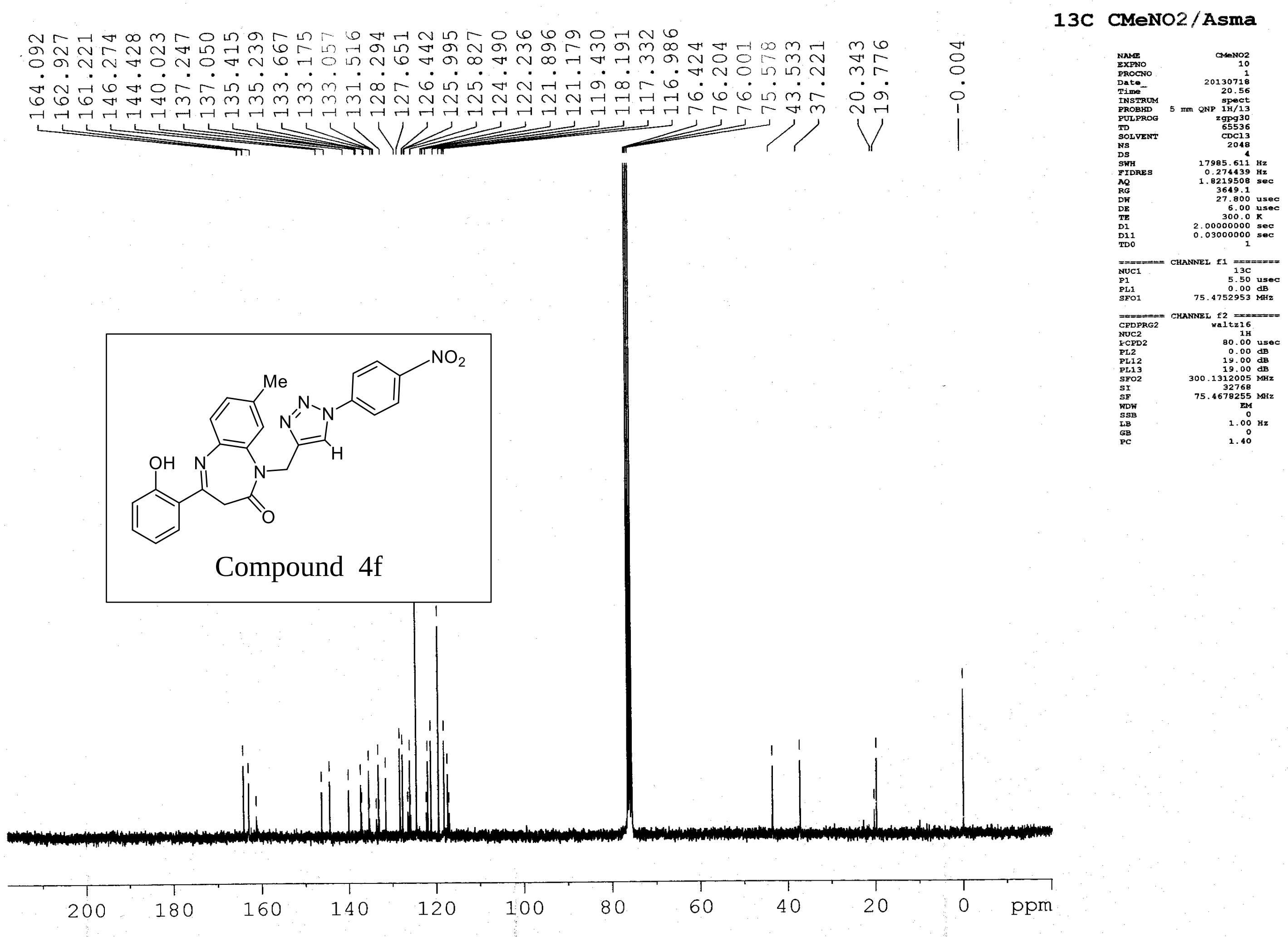

Figure S24. NMR spectra ^{13}C (75,47 MHz, $CDCl_3$) of compound **4f**

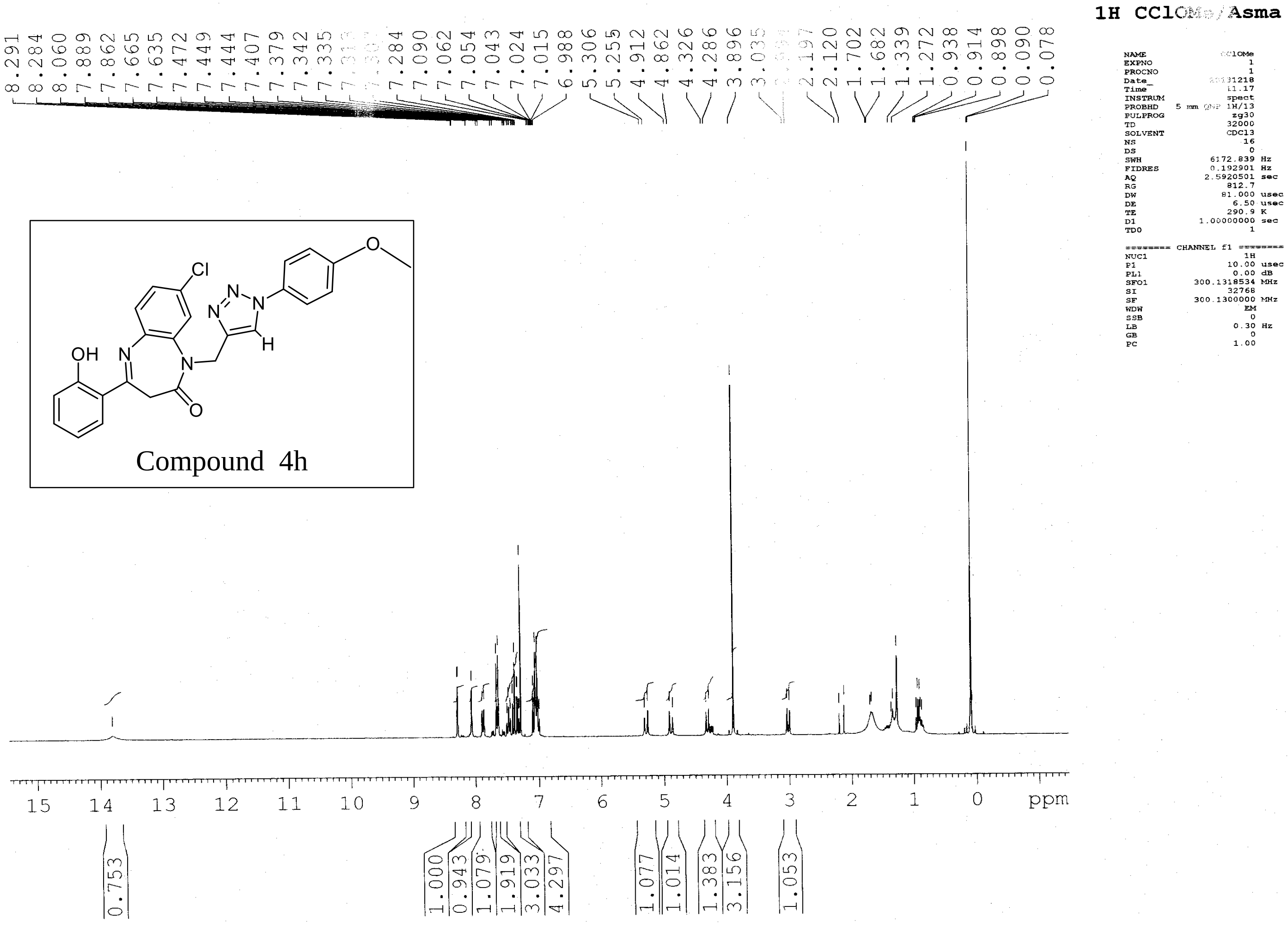

Figure S25. NMR spectra ^{1}H (300 MHz, $CDCl_3$) of compound **4h**

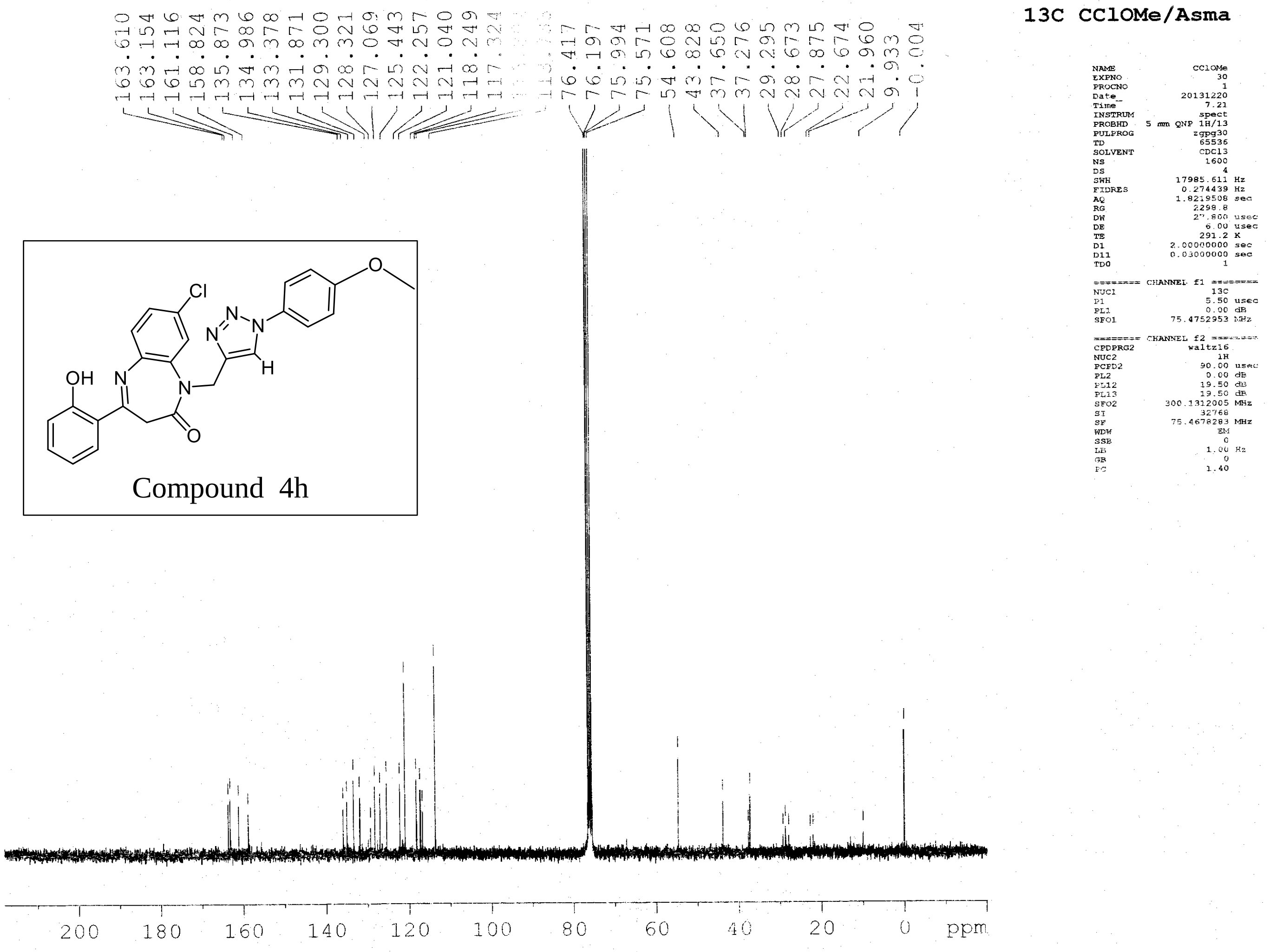

Figure S26. NMR spectra ^{13}C (75,47 MHz, $CDCl_3$) of compound **4h**

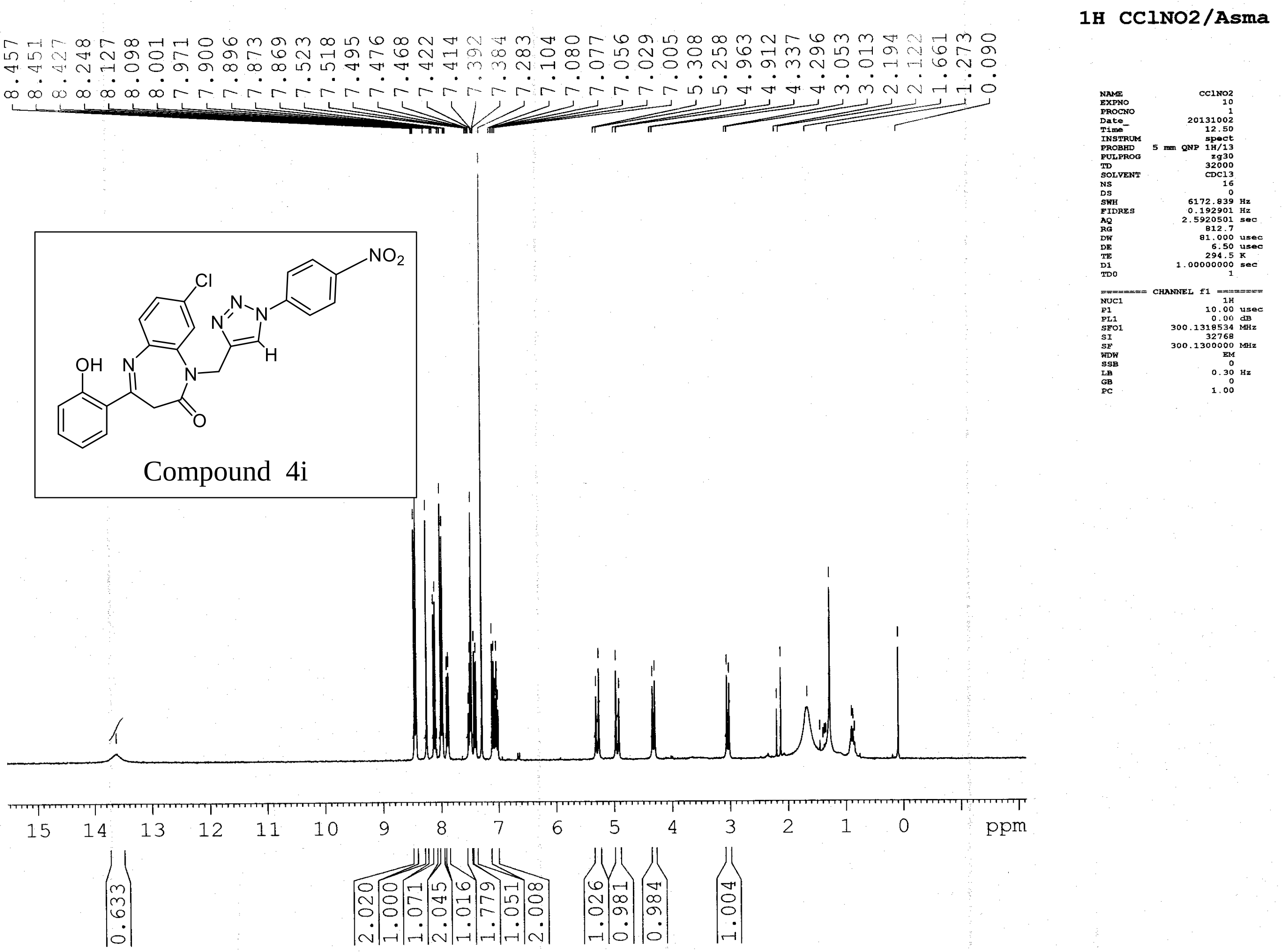

Figure S27. NMR spectra ^{1}H (300 MHz, $CDCl_3$) of compound **4i**

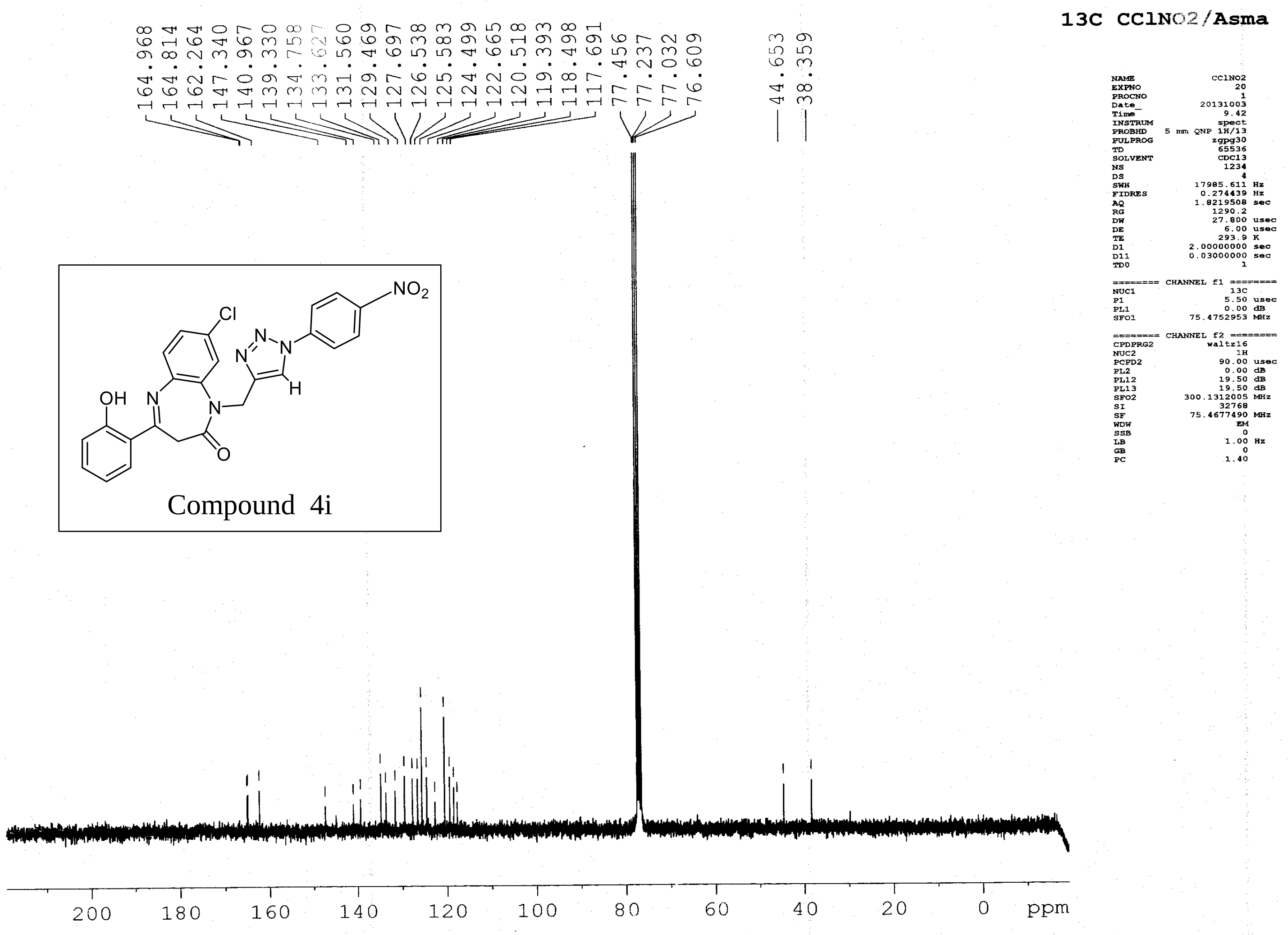

Figure S28. NMR spectra ^{13}C (75,47 MHz, $CDCl_3$) of compound **4i**
